# An integrative analysis of cardiac autonomic neuropathy and nephropathy risk assessed with SUDOSCAN in individuals with type 2 diabetes

**DOI:** 10.3389/fendo.2026.1712085

**Published:** 2026-02-13

**Authors:** Claudiu Cobuz, Mădălina Ungureanu-Iuga, Maricela Cobuz

**Affiliations:** 1 Faculty of Medicine and Biological Sciences, Ştefan cel Mare University of Suceava, Suceava, Romania; 2 Integrated Center for Research, Development and Innovation in Advanced Materials, Nanotechnologies, and Distributed Systems for Fabrication and Control (MANSiD), Ştefan cel Mare University of Suceava, Suceava, Romania; 3 “Sfântul Ioan cel Nou” Emergency Clinical Hospital, Suceava, Romania

**Keywords:** artificial neural network, diabetes mellitus, electrochemical skin conductance, prediction, type 2 diabetes

## Abstract

**Introduction:**

The use of non-invasive, rapid screening methods to detect diabetes mellitus complications, such as neuropathy, is a growing trend in modern medicine. This study aimed to investigate the relationship between SUDOSCAN-derived Cardiac Autonomic Neuropathy (CAN) and Nephropathy (Nephro) scores in individuals with type 2 diabetes mellitus and to evaluate the potential of artificial neural networks in predicting these scores.

**Methods:**

A cross-sectional study was conducted, and 150 individuals were included in the statistical analysis to determine the risk of CAN and nephropathy in individuals with type 2 diabetes mellitus using the SUDOSCAN device. The relationships between SUDOSCAN-derived scores and covariate factors (age, sex, diabetes duration, and body mass index) were established through Spearman correlations, a general linear model, and an artificial neural network (ANN).

**Results:**

The results indicated that individuals with diabetes are at higher risk of both cardiac autonomic neuropathy and nephropathy, which are strongly interconnected, mainly due to factors like age, BMI, and blood pressure rather than traditional glycemic markers. A strong inverse correlation was observed between CAN and nephropathy scores (r = -0.83, *p* < 0.05), highlighting a shared mechanism such as endothelial dysfunction and metabolic stress. The CAN score model showed slightly better predictive performance (RMSE 5.36, MAE 4.11) than the nephropathy model (RMSE 5.91, MAE 7.55), while artificial neural networks achieved outstanding classification performance (AUC ≥ 0.97).

**Discussion:**

When used together, the highly sensitive CAN model can be employed for initial screening to prevent missing cases, while the highly-specific Nephro model can confirm risk and minimize false positives, thereby creating an optimal two-step risk stratification strategy. Thus, ANN-based systems can assist clinicians in guiding decisions by prioritizing individuals for further testing, tailoring treatments, and optimizing follow-up care in diabetic nephropathy.

## 1 Article types

Original Research.

## 2 Introduction

Diabetes mellitus is a chronic metabolic disorder that has gained epidemic dimensions worldwide, posing an essential challenge to global healthcare due to its associated morbidity and mortality. The prevalence of type 2 diabetes mellitus is rising globally (1). Complications stemming from diabetes are a principal cause of disease and death, accounting for substantial healthcare costs (2).

Among the many complications of diabetes mellitus, Cardiovascular Autonomic Neuropathy (CAN) is noted as one of the most insidious and least recognized (1). CAN reflects a progressive process characterized by damage to autonomic nerve fibers innervating the heart and blood vessels, leading to abnormal heart rate control and vascular dynamics (3). CAN is defined as the impairment of autonomic control of the cardiovascular system. It is a common and serious complication of both type 1 and type 2 diabetes, and it significantly increases mortality and morbidity rates (4). The prevalence of CAN increases substantially with diabetes duration (5). In type 2 diabetes, prevalence rates as high as 65% were established in cohorts of individuals with high diabetes duration, and one multicenter study found that 88% of individuals with long diabetes duration (type 1 and type 2) were diagnosed with CAN (1, 3).

Concurrently, diabetic nephropathy or diabetic kidney disease, stands as a major microvascular complication and is one of the leading causes of end-stage renal disease and death in diabetic individuals (2). Diabetic kidney disease significantly increases both all-cause and cardiovascular mortality (4). Early detection of individuals at risk of developing diabetic kidney disease is challenging, as chronic kidney disease is often asymptomatic until a significant portion of kidney function has declined (6).

There is a substantial and critical link between these two complications in type 2 diabetes mellitus. CAN is a strong, independent predictor of rapid kidney function decline in both type 1 and type 2 diabetes mellitus (4). The presence of CAN is associated with increased odds of rapid kidney function decline in type 2 diabetes mellitus (4). Furthermore, diabetic nephropathy is observed significantly more often in individuals with diagnosed CAN compared to those without. In addition, individuals with CAN often present with elevated creatinine levels (1). One hypothesized mechanism linking these conditions is that autonomic dysfunction involves sympathetic activation, which may lead to constriction of renal pre-glomerular vessels, contributing to proteinuria and consequent deterioration of kidney function (3).

Risk factors frequently associated with both the development and progression of CAN and diabetic nephropathy in type 2 diabetes mellitus include older age, longer diabetes duration, hypertension (high systolic blood pressure), and metabolic derangements such as poor glycemic control (HbA1c) and dyslipidemia (e.g., elevated triglycerides) (1–3, 7). Obesity is also associated with impairment of cardiac autonomic function in individuals with type 2 diabetes mellitus individuals (8). Given that CAN independently predicts diabetic kidney disease progression, the identification of CAN through readily available measures may improve the identification of type 2 diabetes mellitus individuals at the highest risk of kidney function loss (4).

The SUDOSCAN device, which non-invasively measures Electrochemical Skin Conductance (ESC), is an essential tool for the early and accessible identification of diabetic nephropathy and CAN (9). ESC assesses sudomotor function, reflecting the integrity of unmyelinated sympathetic C-fibers, which are vulnerable to damage from metabolic processes associated with diabetes (10). For diabetic kidney disease screening, ESC measured by SUDOSCAN is strongly related to parameters of kidney disease, including reduced estimated glomerular filtration rate (eGFR) and albuminuria in individuals with type 2 diabetes mellitus (9, 11). This association is important because many individuals develop diabetic kidney disease without albuminuria, making ESC useful for detecting reduced eGFR (9). The device incorporates built-in algorithms to generate a nephropathy score (SUDOSCAN-Nephro score), with a lower score indicating an increased risk of progression to severe chronic kidney disease requiring dialysis, highlighting its potential predictive value (12). In terms of CAN, ESC non-invasively assesses peripheral autonomic nerve function, and the device provides a dedicated CAN risk score (SUDOSCAN-CAN score) by integrating ESC with patient-specific variables, such as age, height, weight, and HbA1c (13). Since SUDOSCAN testing is quick, simple, reproducible, and comparatively low-cost, it serves as an effective strategy to screen large populations with type 2 diabetes mellitus rapidly, identifying high-risk individuals who should be referred for more costly and specific renal/cardiological (or neurological) evaluations, making it highly useful in low-resource or outpatient settings.

Some studies investigated the relationships between CAN and nephropathy in diabetic individuals. A study in type 1 diabetes mellitus found that the presence of CAN was associated with a 7.8% higher albuminuria increase per year, suggesting that CAN acts as a potential marker for diabetic kidney disease progression (14). Furthermore, this association was driven primarily by measures of sympathetic dysfunction (14). In a meta-analysis concerning type 1 diabetes mellitus risk factors, CAN was significantly associated with microalbuminuria, indicating that individuals with microalbuminuria face a threefold increased risk of developing CAN (7). Mechanistically, CAN may accelerate kidney damage because the resulting sympathetic overactivation leads to higher blood pressure and increased glomerular pressure (14, 15). A review pointed out that CAN significantly impacts the incidence and progression of diabetic kidney disease in individuals with diabetes by promoting kidney hemodynamic imbalance, loss of nocturnal blood pressure dipping, and increased oxidative stress (16). However, there is a gap in the literature regarding the relationship between CAN and Nephro – derived scores assessed by SUDOSCAN in type 1 diabetes mellitus individuals. This study aimed to assess the potential of SUDOSCAN – derived scores, combined with an artificial neural network (ANN), as a screening tool applicable in routine practice. individuals.

## 3 Materials and methods

### 3.1 Cohort characteristics

A cross-sectional study design was used. A total of 170 adult individuals from the Department of Diabetes, Nutrition, and Metabolic Diseases of “Sfântul Ioan cel Nou” Clinical Hospital in Suceava, Romania, were selected for this study. After outliers’ removal, 150 individuals were included in the statistical analysis. The study sample size was determined based on the total population of 35217 individuals, using a 95% confidence level, and a margin of error of 8%. This sample size provides sufficient precision to estimate proportions in the population while maintaining adequate statistical power for detecting moderate effect sizes in our analyses. Based on an estimated difference of 15% in CAN and nephropathy prevalence between at-risk and not-at-risk participants, the study achieves approximately 84% power at a two-sided significance level of 0.05. Data was collected between October 2024 and September 2025. The exclusion criteria was: type 1 diabetes mellitus, age < 18 years, pregnant or breastfeeding females, presence of limb amputation, presence of pacemakers or other implantable electronic devices, open or infected wounds on the hands or feet, history of epilepsy or active seizure disorders, and presence of only one risk (only CAN or only Nephro risk according to SUDOSCAN). The individuals who did not consent to participate in this study were also excluded. Individuals, irrespective of their symptomatic presentation of nephropathy and cardiac autonomic neuropathy, were included in this study.

### 3.2 Physical and laboratory measurements

A standardized medical evaluation was conducted for each participant by trained physicians. The study collected some demographic and clinical information, such as age, sex, type and duration of diabetes, body mass index (BMI), and current medications. Resting systolic and diastolic blood pressure (BP) were measured after 5 minutes in the supine position.

Laboratory evaluations included the quantification of glycated hemoglobin (HbA1c), total cholesterol, low-density lipoprotein (LDL) cholesterol, triglycerides, and serum creatinine concentrations. All samples were analyzed using certified clinical analyzers located in the same hospital department as described in the study’s protocol.

### 3.3 SUDOSCAN measurements

Peripheral sweat gland function was evaluated using the SUDOSCAN device (Impeto Medical, Paris, France) to assess small fiber neuropathy and autonomic function. This non-invasive, non-preparatory test utilizes chronoamperometry and reverse iontophoresis (17). Participants were instructed to refrain from applying lotions, creams, or emollients to their hands and feet prior to the procedure. The patient placed their palms and soles on large stainless-steel electrodes for two minutes. A low-voltage direct current (< 4V) was applied, inducing an electrochemical reaction between the sweat chloride ions and the electrodes (18). The device measures the resultant current to calculate the Electrochemical Skin Conductance (ESC), which is expressed in micro-Siemens (μS). ESC values directly reflect sweat gland chloride ion flow, which is a proxy for the function of small, unmyelinated sympathetic C-fibers (10).

The SUDOSCAN device also provided two additional risk scores: a nephropathy risk score (Nephro score) and a cardiac autonomic neuropathy (CAN) risk score. These scores, automatically calculated by the device’s algorithms, integrated the ESC values with patient data, including age, height, weight, sex, and BMI. The study used the scores directly from the device output without modification. The neuropathy risk score, expressed on a scale from 0 to 150, indicates a higher risk of small fiber/autonomic neuropathy with lower values. According to the manufacturer, a score ≥ 70 is considered normal (no risk), whereas a score < 70 suggests an increased risk of neuropathy. However, in this study, a previously established cutoff value for the Nephro score of 60 was considered (11). For the CAN score, a score < 25 indicates no risk, while values > 25% suggest the presence of CAN risk (19).

Individuals were clustered in two groups: G1 – not at risk of CAN and Nephropathy, which presented a CAN score < 25 and a Nephro score > 60; G2 – at risk of CAN and Nephropathy, with a CAN score > 25 and a Nephro score < 60. These cut-off values were chosen based on our previous study (11) and given that our focus is on risk stratification within the study design, rather than diagnostic confirmation.

### 3.4 Statistical processing of data

Data was processed using SPSS trial version software and XL STAT (2024 version). The differences among groups were tested using the Mann-Whitney test for non-normally distributed data. Spearman correlations were used along with Principal Component Analysis for relationship evaluation. The mathematical modeling of CAN and Nephro scores was done through a general linear model, and the influence of factors was considered significant at p < 0.05. An Artificial Neural Network (ANN) Multilayer Perceptron was applied for CAN and Nephro score risk modelling. The best models were selected based on the lowest sum of square errors. The performance of ANN models was evaluated using Root Mean Square Error (RMSE) and Mean Absolute Error (MAE), as well as Receiver Operating Characteristic (ROC) analysis (Equations 1, 2, 3, 4, 5, 6, 7). Missing data were estimated using the MCMC multiple imputation algorithm. The system used for data processing was powered by an Intel^®^ Core™ i7-1065G7 processor running at 1.30 GHz, with 4 GB of RAM, a 238 GB solid-state drive, and Intel^®^ Iris^®^ Plus Graphics (128 MB). To guarantee ANN reproducibility, the default random seed in SPSS was kept constant across all runs. Data preprocessing, model construction, and validation were performed entirely within the SPSS environment. In addition, ANN analyses were executed repeatedly to verify robustness, yielding consistent and stable results.

(1) $$Accuracy\;(\%)=\frac{TP+TN}{FP+FN+TP+TN}\times 100\;$$

(2) $$Sensitivity\;(\%)=\frac{TP}{FN+TP}\times 100\;$$

(3) $$Specificity\;(\%)=\frac{TN}{FP+TN}\times 100$$

(4) $$PPV\;(\%)=\frac{TP}{FP+TP}\times 100$$

(5) $$NPV\;(\%)=\frac{TN}{FN+TN}\times 100$$

(6) $$AUC=\sum_{i=1}^{n-1}\sum (FPR{}_{i+1}-FPR_{i})\times \frac{TPR_{i+1}+TPR_{i}}{2}$$

where:

(7) $$TPR=\frac{TP}{FN+TP},\;\;\;FPR=\frac{FP}{TN+FP}$$

TP = True Positives, TN = True Negatives, FP = False Positives, FN = False Negatives.

## 4 Results

### 4.1 Characteristics of the cohort

The G1 group, comprising individuals at no risk, accounted for 40.67% of the total patient population. In contrast, the second group (G2), which consists of individuals at risk for CAN and nephropathy, accounted for the remaining 59.33% (**Table 1**). A percentage of 61.33% of the individuals were male, and 38.67% were female. The patient population was distributed across various age ranges and durations of diabetes, with no single group being overwhelmingly dominant. The most significant proportion of individuals (39.33%) had a diabetes duration of 6–15 years, while the remaining participants were almost equally split between those with a duration of 0–5 years and those with a duration greater than 15 years. Similarly, the cohort’s age was diverse, with the 61–70 years group representing the main segment (31.33%), followed by the other three age brackets, which each accounted for 20-25% of the total. Overall, these findings suggest that the study included a balanced and heterogeneous patient group, which is crucial for the generalizability of the results.

**Table 1:** Socio-demographic characteristics of the groups.

Variable	Frequency	Percent (%)
Group		
G1 (CAN score < 25, Nephro score > 60)	61	40.67
G2 (CAN score > 25, Nephro score < 60)	89	59.33
Sex		
Men	92	61.33
Women	58	38.67
Diabetes duration (years)		
0-5	46	30.67
6-15	59	39.33
> 15	45	30.00
Age (years)		
< 50	36	24.00
50-60	38	25.33
61-70	47	31.33
> 70	29	19.33

The characteristics of the two groups are presented in **Table 2**. It can be observed that the G1 group has significantly higher diabetes duration and BMI values compared to the G2 group (*p* < 0.05). The group at risk of CAN and nephropathy (G2) had lower ESC values for both feet and hands, as well as a lower Nephro score, compared to the G1 group. In contrast, the triglycerides, SBP, and CAN score were significantly higher (*p* < 0.05) in G2 compared to G1.

**Table 2:** Differences among groups without (G1) and with (G2) CAN and Nephro risk.

Variable	Mean		Median		Std. error of mean	
	G1	G2	G1	G2	G1	G2
Age	43.75	66.85	47.00^b^	67.00^a^	1.70	0.73
ESC rf	83.80	66.20	85.00^a^	70.00^b^	0.76	1.70
ESC lf	83.31	66.56	85.00^a^	71.00^b^	0.76	1.65
ESC lh	72.03	58.98	74.00^a^	63.00^b^	1.50	1.61
ESC rh	71.97	57.18	74.00^a^	60.00^b^	1.46	1.62
Diabetes duration	9.66	12.02	9.00^b^	11.00^a^	1.01	0.83
BMI	27.28	30.79	27.00^b^	30.00^a^	0.63	0.61
HbA1c	8.85	8.24	8.30^a^	8.17^a^	0.31	0.17
Total cholesterol	171.88	169.61	163.00^a^	168.00^a^	5.58	4.50
Triglycerides	126.56	178.23	81.00^b^	120.00^a^	16.02	17.09
LDL cholesterol	113.76	111.07	113.54^a^	110.00^a^	5.07	3.74
SBP	130.74	148.22	128.00^b^	143.00^a^	2.32	2.05
DBP	78.57	81.91	78.00^a^	81.00^a^	1.31	1.15
Creatinine	0.85	0.88	0.78^a^	0.84^a^	0.06	0.03
CAN score	13.69	39.57	15.00^b^	39.00^a^	1.16	0.77
Nephro score	90.95	49.06	89.00^a^	52.00^b^	2.00	0.92

ESC, Electrochemical skin conductance; rf, right foot; lf, left foot; rh, right hand; lh, left hand; BMI, body mass index; SBP, systolic blood pressure; DBP, diastolic blood pressure; CAN, cardiac autonomic nephropathy; Nephro, nephropathy; a, b, medians followed by different letters in the same row indicate significant differences among groups (p < 0.05) according to the Mann-Whitney test.

### 4.2 Relationships between variables

Spearman correlation coefficients are presented in **Table 3**.

**Table 3:** Spearman correlations between variables.

Variable	Age	Sex	ESC rf	ESC lf	ESC lh	ESC rh	Diabetes duration	BMI	HbA1c	Total cholesterol	Triglycerides	LDL cholesterol	SBP	DBP	Creatinine	CAN score	Nephro score
Age	1.00																
Sex	0.00	1.00															
ESC rf	-0.38^**^	0.10	1.00														
ESC lf	-0.36^**^	0.10	0.93^**^	1.00													
ESC lh	-0.36^**^	0.22^**^	0.50^**^	0.55^**^	1.00												
ESC rh	-0.39^**^	0.23^**^	0.54^**^	0.58^**^	0.93^**^	1.00											
Diabetes duration	0.12	0.08	-0.08	-0.12	-0.10	-0.08	1.00										
BMI	0.27^**^	0.05	-0.18^*^	-0.13	-0.07	-0.07	-0.17^*^	1.00									
HbA1c	-0.19^*^	-0.02	0.02	-0.05	-0.02	-0.01	0.23^**^	-0.12	1.00								
Total cholesterol	-0.08	0.04	-0.06	-0.06	0.00	0.01	-0.10	-0.05	0.10	1.00							
Triglycerides	0.22^**^	-0.05	-0.17^*^	-0.20^*^	-0.12	-0.13	-0.08	0.27^**^	0.17^*^	0.34^**^	1.00						
LDL cholesterol	-0.05	-0.05	0.00	0.02	0.00	-0.01	-0.12	-0.06	-0.02	0.75^**^	0.26^**^	1.00					
SBP	0.44^**^	-0.07	-0.22^**^	-0.16^*^	-0.08	-0.08	-0.07	0.49^**^	-0.06	0.04	0.20^*^	-0.03	1.00				
DBP	0.14	-0.07	-0.08	-0.07	-0.14	-0.10	-0.17^*^	0.32^**^	0.05	0.13	0.17^*^	0.00	0.65^**^	1.00			
Creatinine	0.29^**^	-0.31^**^	0.09	0.09	-0.08	-0.07	0.03	0.12	-0.14	-0.18^*^	0.19^*^	-0.12	0.14	0.07	1.00		
CAN score	0.67^**^	-0.08	-0.69^**^	-0.64^**^	-0.42^**^	-0.45^**^	0.09	0.56^**^	-0.11	0.02	0.31^**^	-0.03	0.50^**^	0.29^**^	0.11	1.00	
Nephro score	-0.85^**^	0.07	0.71^**^	0.70^**^	0.49^**^	0.53^**^	-0.17^*^	-0.22^**^	0.10	-0.01	-0.26^**^	-0.02	-0.33^**^	-0.06	-0.16	-0.83^**^	1.00

** correlation is significant at the 0.01 level (2-tailed), * correlation is significant at the 0.05 level (2-tailed), ESC, Electrochemical skin conductance; rf, right foot; lf, left foot; rh, right hand; lh, left hand; BMI, body mass index; SBP, systolic blood pressure; DBP, diastolic blood pressure; CAN, cardiac autonomic nephropathy; Nephro, nephropathy.

A weakly significant negative correlation was observed between all the ESC values and age (*p* < 0.05, r < -0.36). Age was positively moderately correlated with SBP (*p* < 0.05, r = 0.44) and strongly correlated with CAN score (*p* < 0.05, r = 0.67). In contrast, the correlation with the Nephro score was powerful and negative (*p* < 0.05, r = -0.85). Some medium-positive correlations were observed among ESC parameters (*p* < 0.05, r > 0.50). All the ESC parameters were medium-strongly negatively correlated with the CAN score (*p* < 0.05, r > -0.42) and positively correlated with the Nephro score (*p* < 0.05, r > 0.49). A weakly significant correlation (*p* < 0.05, r = 0.23) was obtained between diabetes duration and HbA1c. Positive weak correlations (*p* < 0.05, r > 0.27) were observed between BMI and triglycerides and DBP, while with SBP and CAN score, the correlation was medium positive (*p* < 0.05, r > 0.49). Total cholesterol was strongly correlated with LDL cholesterol (*p* < 0.05, r = 0.75) and weakly with triglycerides (*p* < 0.05, r = 0.34). Triglycerides were weakly associated with the CAN score (*p* < 0.05, r = 0.31) in a positive manner and with the Nephro score (*p* < 0.05, r = -0.33) in a negative manner. SBP was medium-strongly correlated with DBP and CAN score (*p* < 0.05, r > 0.50), while DPB has a weak positive correlation with CAN score (*p* < 0.05, r = 0.29). A strong negative correlation was observed between CAN and Nephro score (*p* < 0.05, r = -0.83).

The contribution of each variable to the total variance is displayed in **Table 4**. Only the principal components with eigenvalues > 1 were considered. PC1 explained 30.89% of data variability, PC2 accounted for 12.25%, PC3 explained 10.99% of the total variance, followed by PC4 with 7.72%, PC5 with 6.90%, and PC6 with 6.46%. 

**Table 4:** Contributions of the principal components to the data variance.

Component	Eigenvalue	Variance (%)	Cumulative variance (%)
PC1	5.56	30.89	30.89
PC2	2.21	12.25	43.14
PC3	1.98	10.99	54.13
PC4	1.39	7.72	61.85
PC5	1.24	6.90	68.74
PC6	1.16	6.46	75.20

The first component was associated with all the SUDOSCAN parameters (**Table 5**). PC2 was associated with the demographic and physical characteristics of the cohort, specifically age, BMI, and SBP. The third principal component (PC3) was associated with total cholesterol, LDL-cholesterol, and DBP. PC4 was related only to sex, while PC5 was associated with HbA1c and creatinine, and PC6 with diabetes duration and triglycerides.

**Table 5:** Component matrix.

Variable	Component					
	PC1	PC2	PC3	PC4	PC5	PC6
Age	-0.77	0.34	-0.07	0.24	0.22	0.09
Sex	0.14	0.17	0.06	0.65	-0.33	0.12
ESC rf	0.75	0.36	0.00	-0.01	0.16	0.09
ESC lf	0.73	0.46	0.01	-0.01	0.19	0.06
ESC lh	0.67	0.43	0.14	0.29	0.05	0.17
ESC rh	0.70	0.46	0.17	0.25	0.04	0.18
Diabetes duration	-0.12	-0.21	-0.34	0.37	-0.21	0.47
BMI	-0.37	0.46	0.22	-0.15	-0.15	-0.01
HbA1c	0.17	-0.47	0.19	-0.16	-0.48	0.47
Total cholesterol	0.03	-0.38	0.81	0.21	0.21	0.06
Triglycerides	-0.16	-0.13	0.26	-0.22	0.19	0.63
LDL cholesterol	0.02	-0.35	0.73	0.18	0.39	-0.14
SBP	-0.47	0.55	0.40	-0.14	-0.23	0.06
DBP	-0.23	0.42	0.48	-0.42	-0.38	-0.01
Creatinine	-0.07	0.16	-0.27	-0.39	0.52	0.43
CAN score	-0.90	0.22	0.05	0.13	0.06	0.03
Nephro score	0.91	-0.10	0.06	-0.24	-0.15	-0.03
Group	-0.88	0.12	0.00	0.18	0.06	0.12

ESC, Electrochemical skin conductance; rf, right foot; lf, left foot; rh, right hand; lh, left hand; BMI, body mass index; SBP, systolic blood pressure; DBP, diastolic blood pressure; CAN, cardiac autonomic nephropathy; Nephro, nephropathy.

**Figure 1:**
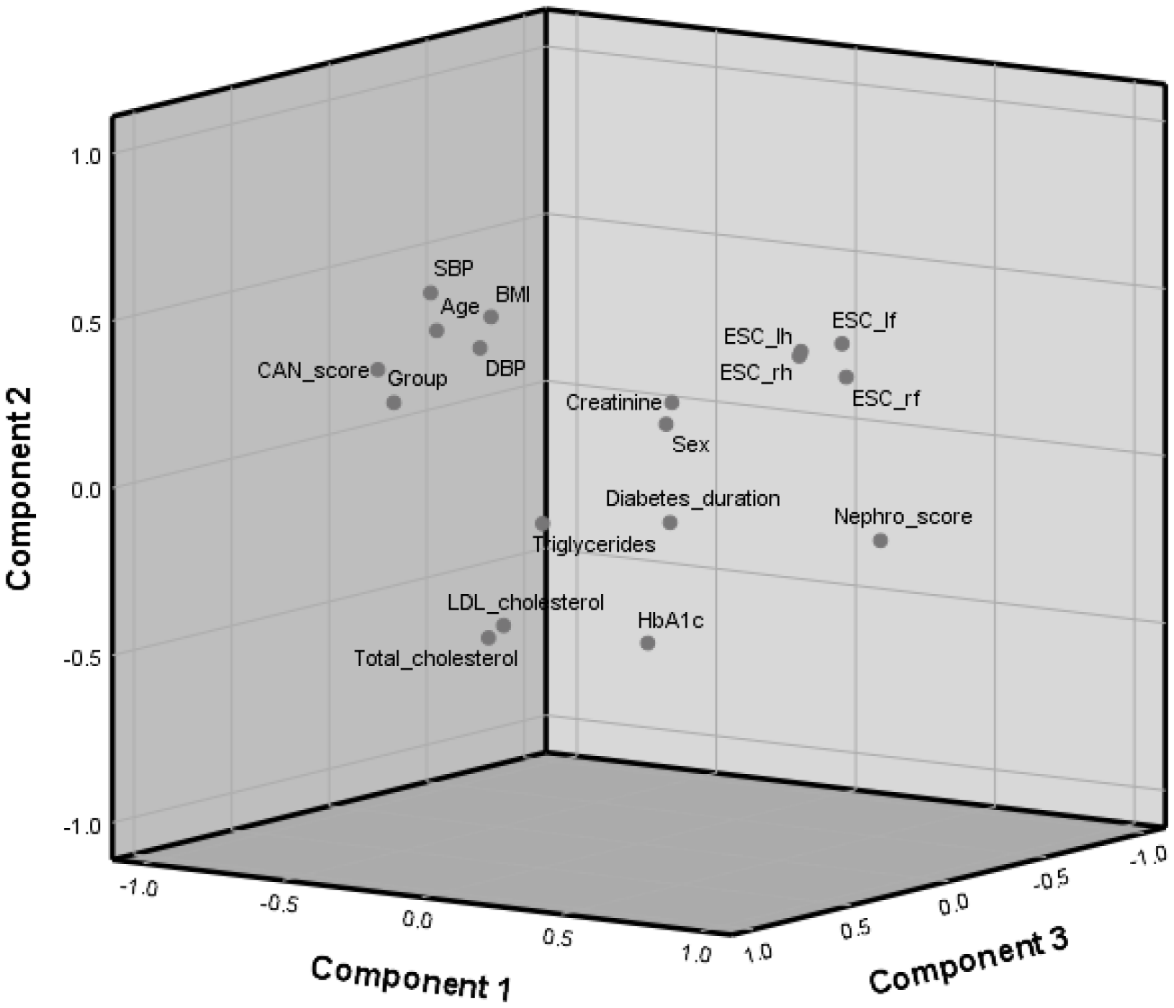
Principal component analysis 3D-plot.

The relationships and associations of the variables with the first principal components are displayed in **Figure 1**. It can be observed the relationships between SUDOSCAN parameters and their opposition with LDL and total cholesterol. The CAN score is positioned in opposition to the Nephro score, a fact confirmed by the negative correlation between them (**Table 3**).

### 4.3 Mathematical modeling of cardiac autonomic neuropathy and nephropathy risk scores

The general linear model proposed for CAN score modelling as a function of CAN score and covariate factors was significant (*p* < 0.05, R^2^ = 0.97), as indicated in **Table 6**. Age, BMI, and Nephro score factors significantly influenced (p < 0.05) the SUDOSCAN CAN score. The highest effect size was observed for Nephro score (partial eta squared = 0.92), followed by BMI (partial eta squared = 0.68).

**Table 6:** ANOVA results of the between-subjects effects for the CAN score.

Factor	T III SS	Degree of freedom	x¯^2^	F-value	*p*-value	Partial η^2^
Corrected Model	32946.52^a^	72	457.59	40.77	0.00	0.97
Intercept	39.23	1	39.23	3.50	0.07	0.04
Age	103.51	1	103.51	9.22	0.00	0.11
BMI	1867.15	1	1867.15	166.37	0.00	0.68
Nephro score	10554.83	70	150.78	13.44	0.00	0.92

a R^2^ = 0.97 (Adjusted R^2^ = 0.95), T III SS, Type III Sum of Squares; x¯^2^, mean squared.

The general linear model used for the Nephro score was also significant (*p* < 0.05, R^2^ = 0.96). All the factors considered – age, BMI, and CAN score exhibited a significant influence (*p* < 0.05) on Nephro score (**Table 7**). The greatest size effect was observed for the CAN score (partial eta squared = 0.83), compared to the other factors of the model.

**Table 7:** ANOVA results of between-subjects effects for Nephro score.

Factor	T III SS	Degree of freedom	x¯^2^	F-value	*p*-value	Partial η^2^
Corrected Model	81557.77^a^	49	1664.44	51.39	0.00	0.96
Intercept	3526.03	1	3526.03	108.86	0.00	0.52
Age	1840.53	1	1840.53	56.83	0.00	0.36
BMI	2088.59	1	2088.59	64.48	0.00	0.39
CAN score	15261.89	47	324.72	10.03	0.00	0.83

a R^2^ = 0.96 (Adjusted R^2^ = 0.94), T III SS, Type III Sum of Squares; x¯^2^, mean squared.

### 4.4 Artificial neural network-based prediction of cardiac autonomic neuropathy and nephropathy risk

**Table 8** summarizes the ANN architectures and error metrics for predicting the CAN score and Nephro score. Both models used one hidden layer with hyperbolic tangent activation and a single output unit with identity activation, with the CAN score including more input factors (age, sex, diabetes duration, BMI), and covariates (HbA1c, Total cholesterol, Triglycerides, SBP, DBP, LDL cholesterol) than the Nephro score (Age, Diabetes duration, BMI, HbA1c, Total cholesterol, Triglycerides, and Creatinine respectively). Training and testing samples were identical for both models, with 117 (83%) and 24 (17%) cases, respectively. Both models achieved relatively low training SSE and relative errors (CAN: 5.63, 0.10; Nephro: 4.73, 0.08), with slightly higher testing SSE and relative errors (CAN score: 3.71, 0.27; Nephro score: 2.08, 0.27), indicating reasonable generalization. Further testing of RMSE and MAE values reveals that the CAN score model predictions are slightly more precise (RMSE 5.36, MAE 4.11) compared to the Nephro score model (RMSE 5.91, MAE 7.55), indicating somewhat higher variability in nephropathy predictions.

**Table 8:** ANN architecture and error summary for CAN and Nephro score.

Feature		CAN score	Nephro score
Case Processing		Training: 117 (83.0%), Testing: 24 (17.0%), Valid: 141 (100.0%), Excluded: 9	Training: 117 (83.0%), Testing: 24 (17.0%), Valid: 141 (100.0%), Excluded: 9
Input Factors		Age, Sex, Diabetes duration, BMI	Age, Diabetes duration, BMI
Covariates		HbA1c, Total cholesterol, Triglycerides, SBP, DBP, LDL cholesterol	HbA1c, Total cholesterol, Triglycerides, Creatinine
Number of Input Units		135	130
Rescaling Method for Covariates		Normalized	Adjusted normalized
Hidden Layers		1	1
Units in Hidden Layer		12	10
Activation Function (Hidden Layer)		Hyperbolic tangent	Hyperbolic tangent
Output Variable		CAN score	Nephro score
Output Units		1	1
Rescaling Method for Output		Standardized	Standardized
Activation Function (Output Layer)		Identity	Identity
Error Function		Sum of Squares	Sum of Squares
Training	SSE	5.63	4.72
	Relative Error (%)	10	8
Testing	SSE	3.71	2.08
	Relative Error (%)	27	28
	RMSE	5.36	5.91
	MAE	4.11	7.55

**Figure 2:**
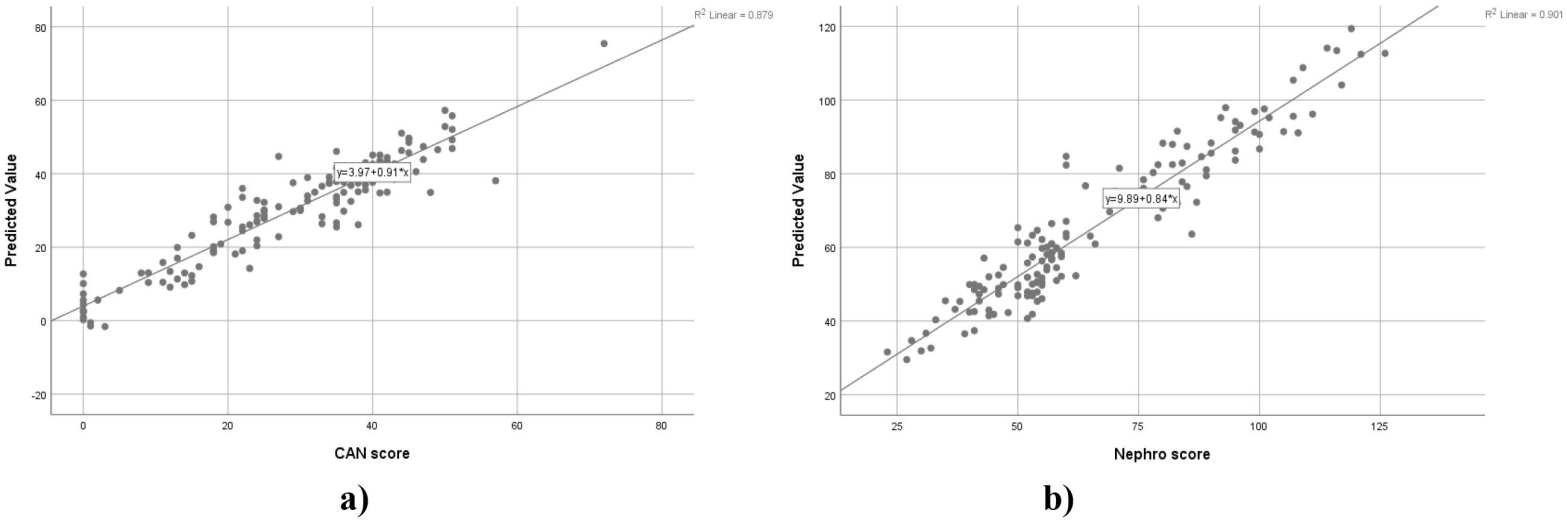
ANN predicted vs. actual values for CAN **(a)** and Nephro score **(b)**.

The graphical representation of the predicted vs. actual values for CAN and Nephro scores is displayed in **Figure 2**. The correlation coefficients were 0.879 for the CAN score and 0.901 for the Nephro score, respectively.

**Figure 3:**
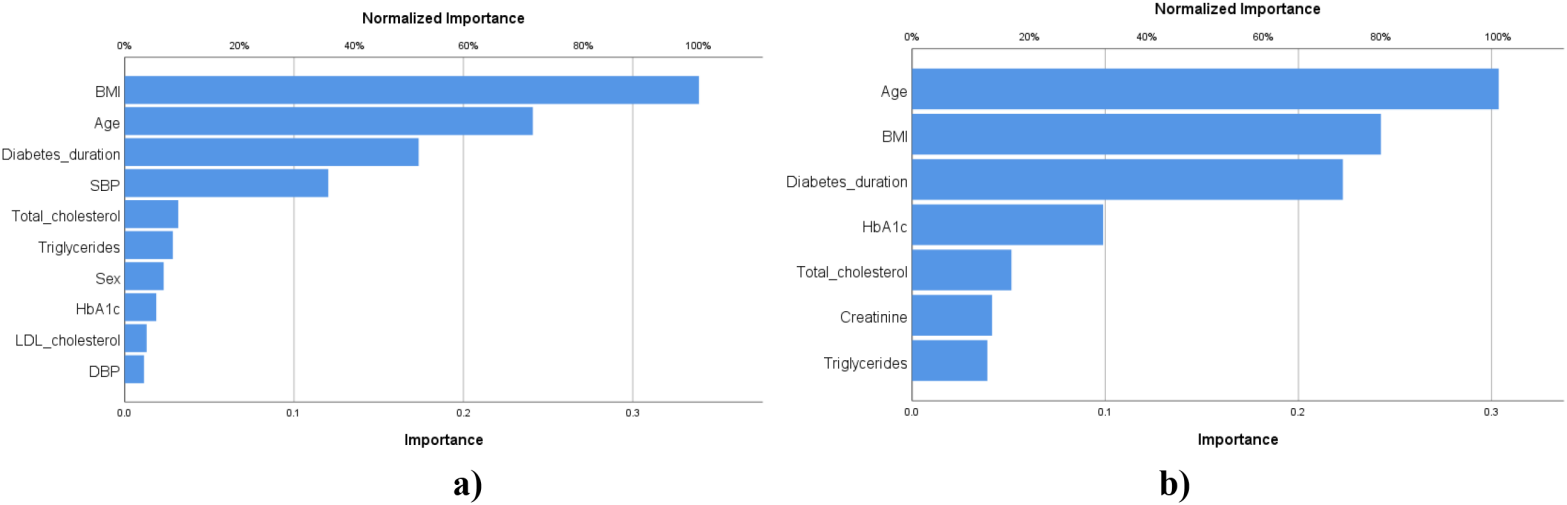
Importance of the factors for CAN **(a)** and Nephro score **(b)** prediction.

According to the results obtained (**Figure 3**), the most important factor for CAN risk prediction is BMI (100% importance), followed by age (71.1%), diabetes duration (51.2%), and SBP (35.5%). For the Nephro score, the age factor presented the highest importance (100%), followed by BMI (79.9%), diabetes duration (73.4%), and HbA1c (32.6%).

The ANN models for CAN score and Nephro score demonstrate excellent performance and are suitable for clinical screening and risk stratification (**Table 9**). Both models show outstanding discriminative ability, with AUCs of 0.97 and 0.98, respectively, indicating they can accurately rank individuals from not-at-risk to at-risk. At the cutoff of 25, the CAN score model achieves very high sensitivity (98.9%) and slightly lower specificity (77.8%), meaning it detects nearly all at-risk CAN individuals while overestimating risk in some not-at-risk individuals, which is clinically safer for screening purposes. In contrast, at the cut-off of 70, the Nephro score model prioritizes specificity (98.2%) over sensitivity (83.3%), ensuring that not-at-risk individuals are rarely misclassified while missing a small proportion of at-risk individuals. Positive and negative predictive values are high for both models, confirming the reliability of predicted risk categories. Cohen’s kappa values of 0.80 for CAN score and 0.79 for Nephro score indicate substantial to almost perfect agreement between the predicted and observed risk categories.

**Table 9:** ANN model performance.

Model	AUC	TP (count)	TN (count)	FP (count)	FN (count)	Accuracy (%)	Sensitivity (%)	Specificity (%)	PPV (%)	NPV (%)	Kappa
CAN score	0.97	86	42	12	1	90.78	98.85	77.78	87.76	97.67	0.80
Nephro score	0.98	70	56	1	14	89.36	83.33	98.25	98.59	80.00	0.79

AUC, area under the curve; TP, True Positives (with-risk correctly predicted); TN, True Negatives (without-risk correctly predicted); FP, False Positives (without-risk predicted as with-risk); FN, False Negatives (with-risk predicted as without-risk).

**Figure 4:**
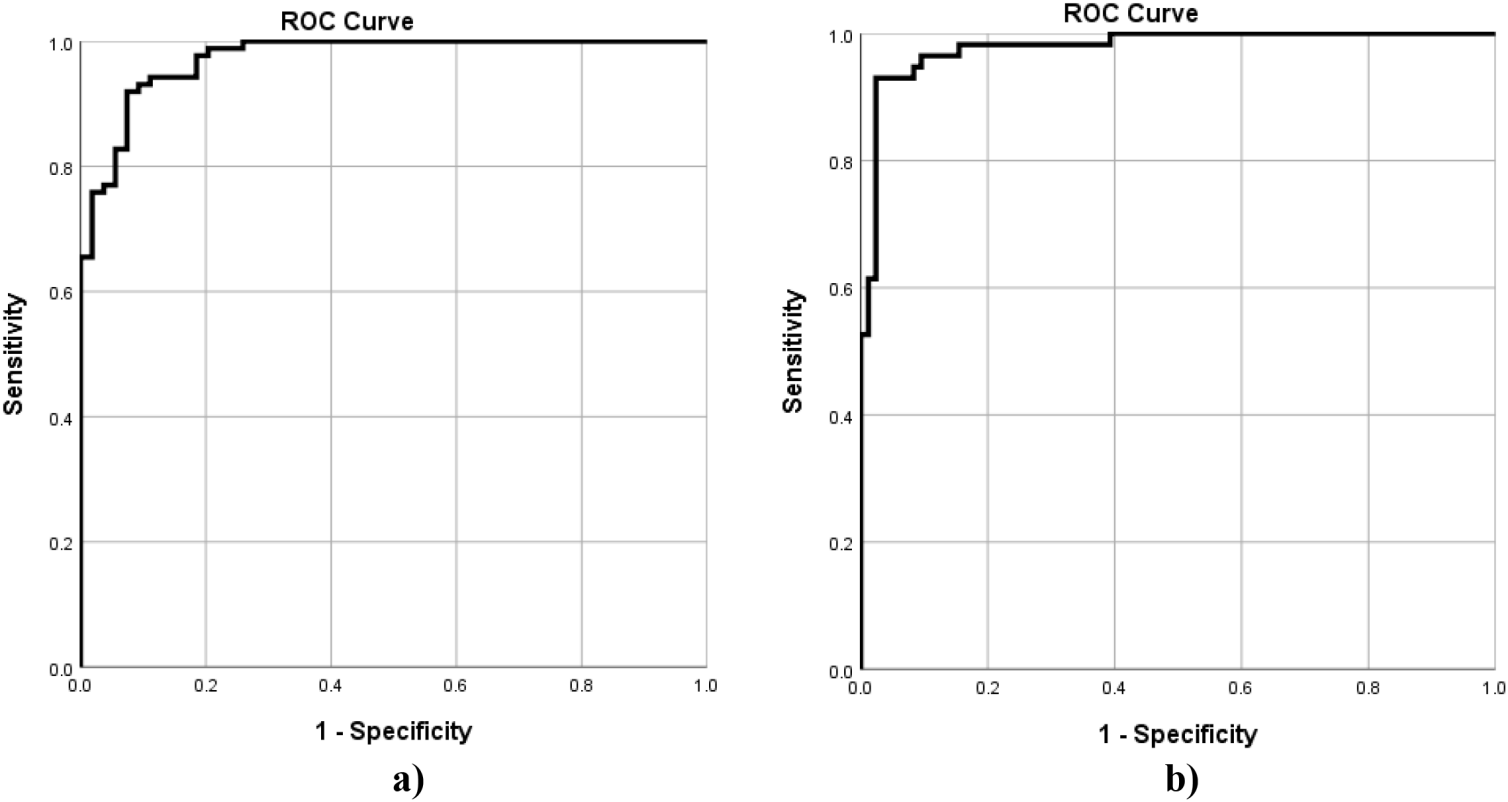
Receiver Operating Characteristic (ROC) Curves for ANN Prediction of CAN **(a)** score Nephro score **(b)**.

The Receiver Operating Characteristic (ROC) curves for CAN and Nephro predicted scores are displayed in **Figure 4**.

## 5 Discussion

The present study confirms that individuals with diabetes mellitus are at increased risk of developing cardiac autonomic neuropathy (CAN) and nephropathy, and that these complications are strongly interconnected. Tang et al. (4) demonstrated that CAN is an important predictor of kidney damage without considering the known diabetic kidney disease factors in individuals with type 2 diabetes. The comparison between at-risk and non-at-risk group revealed significant differences in age, diabetes duration, body mass index (BMI), blood pressure profile, and SUDOSCAN-derived scores. It has been demonstrated that heart rate variability is an important predictor of cardiovascular disease in individuals with diabetic kidney disease (20). Previous studies revealed that the prevalence of CAN is positively correlated with patient age, the duration of diabetes, and the presence of comorbidities such as obesity, smoking, and suboptimal glycemic control (8, 21).

Interestingly, traditional glycemic control markers such as HbA1c, total cholesterol, and LDL cholesterol did not significantly differentiate the groups, highlighting the predominant role of extraglycemic factors in the progression of micro- and macrovascular complications. A meta-analysis study in individuals with type 1 diabetes also revealed that no significant association was found between CAN and cholesterol level, LDL, or HDL levels (7). Another paper discussed the limitations of HbA1c as a marker for glycemic control in individuals with chronic kidney disease, suggesting that alternative markers may be more accurate in this population (22).

The strong inverse correlation observed between CAN and Nephro scores (r = -0.83, p < 0.05) suggests a close interrelationship between autonomic cardiac and renal dysfunction, likely reflecting shared mechanisms such as endothelial dysfunction and chronic metabolic stress. Principal component analysis further confirmed this antagonism, while clinical factors such as age, BMI, and blood pressure emerged as major drivers of variability. Sympathetic nervous system over-activation, a key feature of cardiac autonomic neuropathy (CAN), is hypothesized to directly contribute to the progression of diabetic kidney disease (3, 4). This is because increased sympathetic tone can lead to harmful effects on the kidneys, such as high SBP and DBP, activation of the renin-angiotensin-aldosterone system, and inflammation (5, 23, 24).

General linear models demonstrated the predictive value of SUDOSCAN scores, in combination with simple clinical variables, for identifying individuals at risk. However, the implementation of artificial neural networks (ANN) provided a far more robust classification, with AUC values close to 1.0. In this study, both ANN models for CAN score and Nephro score demonstrated robust performance and generalizability. Training and testing errors were low for both models, indicating good model fitting without substantial overfitting. Standard separation between training and evaluation was followed to minimize the risk of overfitting. The results represent internal performance within the defined cohort and should be interpreted accordingly. The CAN score model showed slightly better predictive precision, with a testing RMSE of 5.36 and MAE of 4.11, compared to RMSE 5.91 and MAE 7.55 for the Nephro score model, reflecting slightly higher variability in nephrological predictions. These results suggest that both models can reliably predict their respective outcomes, with CAN score predictions being marginally more consistent. The differences in sensitivity and specificity highlight their complementary clinical utility: the CAN model exhibited very high sensitivity, making it suitable as a screening tool, minimizing the risk of missed cases; the Nephro model displayed very high specificity, making it appropriate for risk confirmation, reducing false-positive classifications. Clinically, the ANN models proposed for CAN and Nephro scores can guide early intervention, with potential cutoff adjustments to balance sensitivity and specificity according to patient safety priorities. Therefore, a combined approach (screening with the CAN model and confirmation with the Nephro model) may represent an optimal risk stratification strategy in clinical practice. Our results are in agreement with those reported by Hosseini et al. (2) who developed a prediction model for diabetic nephropathy in type 2 diabetic individuals, and identified age as the most important factor, followed by the duration of diabetes, systolic blood pressure, and body mass index. Sabanayagam et al. (6) also utilized machine learning to predict the risk of diabetic nephropathy, and the model proposed identified age, BMI, diabetes duration, and SBP as important predictors of nephropathy risk. Leet et al. (1) and Lee et al. (25) stated that the prevalence of CAN was high in individuals with high diabetes duration and observed some key risk factors such as long diabetes duration, deficient glycemic control, hypertension, higher creatinine levels, and the presence of other microvascular complications.

The limitations of this study include the relatively small sample size and the absence of additional biomarkers (e.g., albuminuria, NT-proBNP), which could have provided further validation of SUDOSCAN-derived scores. Moreover, due to its cross-sectional design, causal relationships between the identified predictors and the progression of diabetic complications cannot be established. In addition, it was a single-center, retrospective study involving a homogeneous ethnic group, which may limit its generalizability. The absence of clinical markers such as albuminuria, proteinuria, eGFR, etc., also represents a limitation of this study, along with the lack of assessment for diabetic sensorimotor polyneuropathy and diabetic retinopathy. Menshov et al. (26) showed that nicotine from different delivery systems (cigarettes, e-cigs, heating devices, oral packs) causes varying degrees of autonomic activation, affecting sympathetic and parasympathetic activity measured by heart rate variability and stress hormone changes. The delivery method of nicotine significantly influences autonomic balance and related physiological functions like sweating (26). Neurotrophic therapies can impact autonomic nervous system function by promoting survival/regeneration of autonomic nerve fibers (27), including those that innervate sweat glands, impacting the sudomotor (sweating) function. In our study, smoking and medication were not included in the ANN model, which may be considered a limitation. This paper presents a model-based observation specific to the present cohort and target outcomes and should not be interpreted as a general causal hierarchy.

The strength of this work lies in the integration of SUDOSCAN measurements for the simultaneous assessment of CAN and nephropathy risk within the same patient cohort. By combining correlation analysis, principal component analysis, and linear regression with advanced ANN modeling, this study provides a comprehensive evaluation of risk patterns, the interdependence between CAN and Nephro scores, and predictive performance. From a practical perspective, the early detection of cardiac autonomic neuropathy and renal impairment is of major clinical importance, as these complications significantly affect cardiovascular outcomes and quality of life in individuals with diabetes. Conventional diagnostic methods, such as autonomic function testing or microalbuminuria measurement, are often invasive, time-consuming, or less feasible for routine screening. In this context, SUDOSCAN represents a rapid, non-invasive, and reproducible tool that can be readily applied in daily clinical practice for assessing cardio-renal risk. The integration of artificial intelligence through neural network models further enhances clinical utility by enabling not only the identification of high-risk individuals but also risk stratification according to severity. This approach allows clinicians to make more informed decisions regarding monitoring, treatment intensification, and referral for additional investigations.

Ultimately, this integrative framework bridges classical statistical methods and machine learning, providing a clinically applicable strategy for patient screening and the prioritization of preventive interventions. Integration of such tools into electronic health records or bedside devices could enhance accessibility, support precision medicine, and ultimately improve patient outcomes.

## 6 Conclusion

SUDOSCAN, combined with simple clinical parameters (age, BMI, blood pressure, and diabetes duration), is a helpful tool for risk stratification of CAN and nephropathy in individuals with diabetes mellitus. Artificial neural networks demonstrated excellent performance (AUC ≥ 0.97), surpassing traditional statistical models and underscoring the potential of artificial intelligence in predictive medicine. The CAN model, with its high sensitivity, is well-suited for initial screening, while the Nephro model, with its high specificity, is more suitable for risk confirmation. The combined use of both models may optimize early detection and guide personalized interventions for preventing cardio-renal complications. Further longitudinal studies, with larger cohorts and standardized biomarkers, are warranted to validate and generalize these findings. In addition, the incorporation of clinical markers for CAN and nephropathy assessment will be considered, along with the evaluation of diabetic sensorimotor polyneuropathy and retinopathy. This research demonstrated that both SUDOSCAN-derived CAN and Nephro scores can be effectively predicted using artificial neural network models, which showed excellent accuracy, sensitivity, and specificity. The strong performance of these models underscores their potential clinical utility, extending beyond traditional statistical approaches.

From a practical standpoint, integrating ANN-based prediction models into medical practice can provide clinicians with a powerful and user-friendly decision-support tool. Such models can be incorporated into routine screening programs for diabetic individuals, enabling rapid and non-invasive identification of those at increased risk of cardiac autonomic neuropathy and nephropathy. By offering reliable stratification according to risk levels, ANN-based systems can help guide clinical decision-making, such as:

- prioritizing individuals for further diagnostic testing (e.g., autonomic function tests, renal biomarkers),
- tailoring treatment strategies to individual risk profiles,
- optimizing follow-up intervals and monitoring intensity, and
- supporting early preventive interventions to reduce long-term complications.

The implementation of ANN-assisted tools into electronic health record systems or bedside devices could further enhance accessibility and clinical adoption. This integrative approach has the potential to improve patient outcomes, optimize resource allocation, and facilitate precision medicine strategies in the management of diabetes-related complications. The findings should be interpreted as exploratory with respect to implementation.

## Data Availability

The raw data supporting the conclusions of this article will be made available by the authors, without undue reservation.
